# Mitochondria in Injury, Inflammation and Disease of Articular Skeletal Joints

**DOI:** 10.3389/fimmu.2021.695257

**Published:** 2021-09-03

**Authors:** James Orman Early, Lauren E. Fagan, Annie M. Curtis, Oran D. Kennedy

**Affiliations:** ^1^Department of Anatomy and Regenerative Medicine and Tissue Engineering Research Group, Royal College of Surgeons in Ireland, Dublin, Ireland; ^2^School of Pharmacy and Biomolecular Sciences and Tissue Engineering Research Group, Royal College of Surgeons in Ireland, Dublin, Ireland; ^3^Department of Mechanical and Manufacturing Engineering, Trinity College Dublin, Dublin, Ireland

**Keywords:** mitochondria, cartilage, osteoarthritis, inflammation, mechanobiology

## Abstract

Inflammation is an important biological response to tissue damage caused by injury, with a crucial role in initiating and controlling the healing process. However, dysregulation of the process can also be a major contributor to tissue damage. Related to this, although mitochondria are typically thought of in terms of energy production, it has recently become clear that these important organelles also orchestrate the inflammatory response *via* multiple mechanisms. Dysregulated inflammation is a well-recognised problem in skeletal joint diseases, such as rheumatoid arthritis. Interestingly osteoarthritis (OA), despite traditionally being known as a ‘non-inflammatory arthritis’, now appears to involve an element of chronic inflammation. OA is considered an umbrella term for a family of diseases stemming from a range of aetiologies (age, obesity etc.), but all with a common presentation. One particular OA sub-set called Post-Traumatic OA (PTOA) results from acute mechanical injury to the joint. Whether the initial mechanical tissue damage, or the subsequent inflammatory response drives disease, is currently unclear. In the former case; mechanobiological properties of cells/tissues in the joint are a crucial consideration. Many such cell-types have been shown to be exquisitely sensitive to their mechanical environment, which can alter their mitochondrial and cellular function. For example, in bone and cartilage cells fluid-flow induced shear stresses can modulate cytoskeletal dynamics and gene expression profiles. More recently, immune cells were shown to be highly sensitive to hydrostatic pressure. In each of these cases mitochondria were central to these responses. In terms of acute inflammation, mitochondria may have a pivotal role in linking joint tissue injury with chronic disease. These processes could involve the immune cells recruited to the joint, native/resident joint cells that have been damaged, or both. Taken together, these observations suggest that mitochondria are likely to play an important role in linking acute joint tissue injury, inflammation, and long-term chronic joint degeneration - and that the process involves mechanobiological factors. In this review, we will explore the links between mechanobiology, mitochondrial function, inflammation/tissue-damage in joint injury and disease. We will also explore some emerging mitochondrial therapeutics and their potential for application in PTOA.

## Introduction

**Figure 1:**
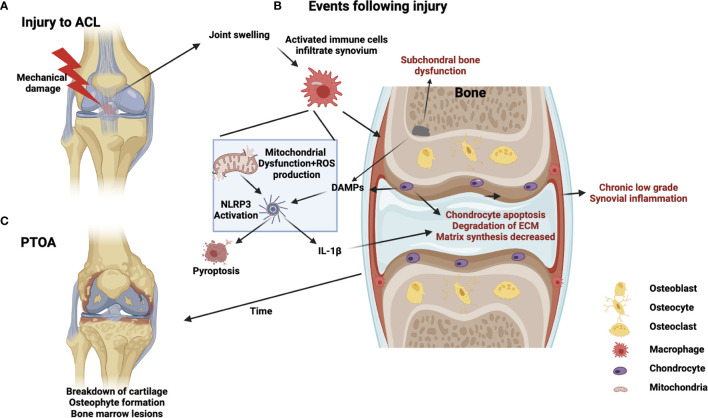
Schematic of PTOA progression illustrating potential mitochondrial involvement in injury, inflammation, and disease. **(A)** Acute injury, such as ACL rupture, results in joint swelling and infiltration of immune cells. **(B)** Crosstalk between immune cells, chondrocytes and subchondral bone cells occurs involving DAMPS, PRRs, mechanotransduction, NLRP3 activation, cell-death/pyroptosis, mitochondrial dysfunction, cytokine release and ROS production from immune cells generating a feed-forward loop to drive chronic inflammation, matrix degradation and subchondral bone dysfunction **(C)** Chronic development of PTOA, long-term low-grade inflammation, bone marrow lesions and cartilage degeneration.

Osteoarthritis (OA) is the most commonly occurring form of joint disease worldwide, affecting approximately 3% of the global population (1). The primary hallmark of OA is the degeneration of articular cartilage, but dysregulation in other joint tissues such as subchondral bone and synovium are also significant factors (2). Joint degeneration results in debilitating stiffness and pain, with major societal and economic implications. Despite the widespread prevalence of OA, and its significant consequences, disease modifying treatments are lacking. Current treatment options are confined to either conservative (physical therapy, exercise, or pain management) or surgical (joint replacement) approaches (3). OA is often associated with aging, but other risk factors such as age, obesity and steroid use exist (4). In addition, acute injury that involves damage of the primary joint tissues (such as the anterior cruciate ligament rupture in the knee), is also a well-established risk factor for joint degeneration – even in relatively young cohorts. The version of disease which develops in this scenario is called Post-traumatic OA (PTOA). PTOA makes up approximately 12% of the overall disease burden of OA and this proportion is set to increase due to increased intensity of exercise/activity being taken up by ever younger age-groups (5). The initial inflammatory response to the injury, driven by a host of molecular and cellular mechanisms, are likely to “set the course” of subsequent disease progression (6). However precisely how the inflammatory response drive resident joint cells into a state of chronic dysfunction is unknown. Interestingly, recent advances in the field suggest a role for altered mitochondrial function as part of the inflammatory response to injury (7), which may be linked to subsequent chronic degeneration. In this review, we will specifically address the links between mitochondrial function, inflammation, and joint disease (**Figure 1**). We will also explore recent advances in mechanobiology and how this may relate to PTOA.

## Joint Cells and Immune Cells Are Involved in PTOA Development

Although many cell types and tissues exist within the joint, it is cartilage and its degeneration which is the central focus of OA (2). Chondrocytes which are the primary resident cells in articular cartilage, are unique in that they exist in an aneural, alymphatic and avascular microenvironment (8). The cartilage extracellular matrix (ECM) is mostly comprised of collagen and proteoglycans (as well as water) (9) all of which are produced by chondrocytes under normal healthy circumstances,. However post-injury, chondrocytes begin to produce enzymes (such as metalloproteases (MMP) and aggrecanases) which are harmful to the ECM (10). This process occurs *via* activation of various pathways such as NF-kB and MAP kinases and is also linked to increased production of Reactive Oxygen Species (ROS), originating from mitochondria (11, 12). These degradative proteases gradually disrupt the collagen network, promoting a loss of proteoglycans and drive degradative changes in the matrix which in turn feeds back to affect chondrocyte health and function (13). Alongside these disruptive changes, the production of collagens and aggrecans, which are required for health homeostasis, are dramatically reduced (14, 15). This switch, whereby chondrocytes reduce production of beneficial ECM proteins, and increase production of harmful enzymes, is thought to involve altered mitochondrial function (16). Injury also has the effect of increasing crosstalk between cartilage and surrounding joint tissues such as subchondral bone and the synovial membrane (17). The latter of which is the central source of acute inflammatory factors as it becomes quickly infiltrated by activated immune cells in response to joint injury (18). Whether immune cells are the initiator of the degenerative switch within the damaged joint or an additional contributor to disease, is not entirely known. Nonetheless it is becoming increasingly clear that immune cells are central to PTOA development. At the cellular level, within the damaged joint, synovial macrophages, fibroblasts and infiltrating monocytes, and T-cells, B-cells, natural killer cells and dendritic cells are all involved in responding to injury and driving local inflammation (19, 20). It now appears likely that crosstalk also occurs between these immune cells and chondrocytes/subchondral bone populations. While the nature of this relationship is unclear it is well established that mitochondrial function governs many aspects of immune function and may play a role in this situation (21, 22). This is especially well documented in macrophages, in which mitochondrial dynamics and function regulate cellular trafficking, cytokine production, phagocytosis and wound repair. While these processes have been well documented in multiple macrophage subtypes, it is yet unclear if they hold true in every population of macrophage that may be involved in PTOA development, such as bone marrow macrophages, osteal macrophages and macrophage like synoviocytes. However, it is conceivable that the mitochondrial changes produce similar effects in these cells and that changes in mitochondrial function influence and drive the development of PTOA after injury. Thus, understanding the details of events that occurs between immune and joint cell-types, in the aftermath of injury, and in particular the role of mitochondria will be crucial in developing new treatments to prevent disease progression.

## Joint Injury, Inflammation and Mechanobiology Have a Role in Development of PTOA

Originally, the process of inflammation was not deemed to be a central factor in the aetiology of OA. Indeed historically it has been known as ‘non-inflammatory OA’, especially when compared to highly inflammatory versions of arthritis such as rheumatoid, psoriatic, and juvenile arthritis. However, it is now established that the persistence of a dysregulated inflammatory response does impact on PTOA (18). Intriguingly, mitochondrial activities have also recently been shown to be significantly modulated by these inflammatory stimuli (23), as well as by other factors such as mechanical stimulus. Therefore, understanding mitochondrial function (including morphology/metabolism) within joint cells in response to injury, may provide new insights in terms of the pathology of PTOA and therapeutic development.

Despite lacking an underlying mechanism to describe the link between mechanical injury and inflammation, it is clear that mechanical joint damage causes migration of immune cells from the circulation through the synovial membrane and into the synovial fluid (19, 20). Injury also causes a certain proportion of cell death in the region. This cell death results in the production of damage associated molecular patterns (DAMPs), which are then detected by Toll-Like Receptor (TLR) proteins on cell surfaces (24). These further promote inflammatory cascades and cell and fluid infiltration and thus a damaging feed-back loop is established. One central mechanism by which DAMPs have been shown to promote inflammation in the joint is *via* activation of the NLRP3 inflammasome in macrophages and subsequent production of IL-1β (25). This mechanism will be discussed in detail below, specifically in the context of joint injury.

## IL-1β Is Central to Tissue Damage, Inflammation and Mitochondrial Responses to Injury

IL-1β is a highly pro-inflammatory cytokine that is involved in a wide variety of disease states and is also increased in cases of OA (26). Specifically, in this scenario, IL-1β is capable of shifting chondrocytes to a catabolic state (27) thus providing a direct link between joint injury, immune cell mitochondria and cartilage degeneration. The specific details of how chondrocytes respond to IL-1β have been studied extensively *in vitro* (27, 28), but importantly it has also been shown to be present in significant quantities in synovial fluid immediately after injury (6) (along with some of its important precursors, such as NLRP3 (29) and other DAMPs [Basic Calcium Phosphate (BSU) (30), monosodium urate (MSU) (31) and ATP (32)]. Intriguingly, IL-1β levels remain elevated for months post-injury (18, 33). Thus, this is likely to be a primary mediator in the pathological process that begins with joint injury and leads to disease. An interesting supporting example of this stems from the large Canakinumab Anti-Inflammatory Thrombosis Outcomes Study (CANTOS), which involved >10,000 patients. CANTOS was a randomized, double-blinded, placebo-controlled trial that investigated the use of canakinumab, a monoclonal antibody targeting IL-1β, on high-risk patients with established atherosclerotic disease who had survived a myocardial infarction (MI) (34). Post-hoc retrospective analyses of this cohort, found that canakinumab treatment was associated with reduced rates of joint replacement as well as OA symptoms. Regulation of IL-1β occurs at both the transcriptional and post-translational levels – and both of these processes have been shown to be mediated by mitochondria which we will detail further below.

Since this cytokine plays such a central role in the response to injury, it seems reasonable to consider it as a potential target for direct OA prevention – following the encouraging data that emerged from the CANTOS trial (34). In fact, treatment strategies that directly target IL-1β and/or its cognate receptor (IL-1R) have been tested (26). However, somewhat surprisingly, these efforts have achieved only limited success. One potential reason for this is that targeting any aspect of the IL-1β pathway, once it has been activated, may be a case of ‘too little, too late’. In order for IL-1β to be released from cells like macrophages it must first be cleaved by an intracellular complex called the inflammasome (25). The inflammasome is a multi-protein complex that responds to both pathogenic micro-organisms and, of direct relevance here, DAMPs which are released within the joint after injury (25). The inflammasome is thus an obligate precursor step to IL-1β activation and most importantly to its release. Targeting inflammasome activation and thus the release of IL-1β may prove to be a more promising therapeutic strategy than targeting released IL-1β in the joint which may already have exerted its catabolic effect on chondrocytes. There are multiple inflammasomes that respond to a host of different signals. However, one in particular called NLRP3 is the most likely candidate for involvement in joint inflammation and responses to DAMP signalling. The NLPR3 inflammasome is composed of (1) a receptor protein called NLRP3, (2) an adaptor molecule called ASC, and (3) Caspase 1 (25). Once NLRP3 senses an activating signal, it oligomerises with ASC and Caspase-1. This activates Caspase-1 allowing it to cleave IL-1β into its active form. This entire process has been shown to be under mitochondrial control. Specifically, high mitochondrial membrane potential (35), mitochondrial ROS (36) and mtDNA (37) are all involved in regulating inflammasome activation. Of equal importance, is the fact that inflammasome activation is also closely linked to the process of pyroptosis, a specific form of inflammatory cell-death - also regulated by mitochondria (38). Pyroptosis also ultimately results in IL-1β release, as well as other inflammatory factors and DAMPs that could potentiate the inflammatory response and drive cartilage degeneration further.

## Multiple Joint Tissues Are Involved in Inflammation After Injury

The source(s) of NLRP3 in the injured joint have not been explicitly identified; however the synovial tissues are an extremely promising candidate - since they have a central role in other examples of joint inflammation. Synoviocytes are the resident cell types of this tissue, (with two sub-types: fibroblasts and ‘Macrophage Like Synoviocytes [MLS]’) and both have been shown to produce high levels of NLRP3 in culture conditions (29, 39). Interestingly, a very recent study has shown that chondrocytes *in vitro* are also capable of expressing inflammasome components and can undergo NLRP3 activation, pyroptosis and IL-1β release when stimulated with LPS and ATP *in vitro* (40). While TLRs are known to be present in chondrocytes, and their expression to be altered in OA (41), the presence of chondrocyte specific inflammasomes is a recent and novel development. These interesting findings must still be balanced against other inflammatory pathways, activated by TNFα for example, which do not act through TLRs, but still increase MMP activity, suggesting that IL-1β may not be the only driver of OA progression. It has also been proposed that even in the presence of NLRP3, ASC and Caspase 1 in chondrocytes, OA cartilage is unable to produce active IL-1β (42). However, these studies used isolated cartilage explant models which lack externally infiltrating immune cells, which are likely to be central to IL-1β release and, thus NLRP3 activity.

Subchondral bone is also known to be involved in the early post-injury phase, evident by the presence of Bone Marrow Lesions (BMLs) in most cases of acute joint injury (17). However, NLRP3 activity has yet to be identified in native bone cells in the osteoblast lineage. Intriguingly however, hydroxyapatite, which is the primary inorganic component of bone tissue, is the predominant form of BCP crystal found in OA joints (43, 44). It is possible that following injury, hydroxyapatite is indirectly released into the joint, following increased subchondral remodelling, (which has been also shown to occur early after injury) or in relation to osteophytes which form later in the disease process. Hydroxyapatite drives NLRP3 activation in macrophages *in vitro* and *in vivo* through potassium efflux and ROS dependant mechanisms (45). Direct delivery of BCP crystals to mouse knees was shown to cause synovial macrophage infiltration, chondrocyte death, synovitis, and cartilage degeneration (46, 47). However, this study concluded that this mechanism was NLRP3 and IL-1 independent. A similar study showed that BCP crystals also impact on macrophage metabolism, creating a shift from oxidative phosphorylation to a glycolytic phenotype, and promoting the expression of the highly pro-inflammatory transcription factor HIF-1α^48^). Thus subchondral bone and its release of hydroxyapatite may be a novel target tissue for indirect modulation of NLRP3 function and macrophage metabolism.

Taken together, these findings suggest that NLRP3 activation, which is tightly regulated by mitochondrial activities, may have an important role in damage responses of multiple joint tissues. Whether there is one critical cellular source leading to NLRP3 activation, or an aggregated combination of many, has yet to be determined. Nonetheless NLRP3, as an obligate gate-keeper of IL-1β activation, potentially in multiple joint tissues, is a prime target in the search for novel therapeutics to limit PTOA.

## Modulating Mitochondrial Responses to Injury as a New Avenue for PTOA Prevention

While the specific aspects of mitochondrial function that relate to NLRP3 and IL-1β production in the joint remain poorly understood, there are some clear contenders for central involvement. For example, mitochondria-derived ROS, which is the predominant source of ROS in cells, is involved in multiple pathologies as well as in the process of ageing (49, 50). A strategy of targeting ROS activity in infiltrating immune, and/or joint, cells could lead to alternative ways to limit catabolic events in the joint post-injury. For example promotion of natural ROS inhibitors *via* antioxidants, falls into this category. Immediately following joint injury and/or cartilage damage, alterations in mitochondrial activity, swelling, polarisation and ROS production occur in chondrocytes (51). The normally hypoxic cartilage microenvironment is quickly altered and chondrocytes become exposed to increased levels of oxygen as well as a variety of other chemical species *via* neovascularisation of subchondral bone and altered synovial activities (52). NRF2 is a master antioxidant factor, which plays an important role in oxidative stress regulation. NRF2 can limit NLRP3 inflammasome activity in macrophages (53) and chondrocytes (40) and thus ultimately inhibit IL-1β activity. NRF2 has also been found to be upregulated in OA patient samples and surgical OA rodent models, suggesting it may have a role in the natural protective response to injury (29).

In addition to the local inflammatory state within the joint, an interesting study showed that the overall inflammatory state of an organism can also affect PTOA development. Priming of mice with lipopolysaccharide (LPS), a major component of bacteria, 5 days prior to joint injury was found to exacerbate the severity of PTOA resulting from injury (53). Subsequent RNA-seq analyses highlighted the same set of genes, that was previously found to be elevated in synovial macrophages from rheumatoid arthritis patients. This suggests the existence of a compounding effect of synovitis along with LPS administration. Furthermore, significantly increased numbers of activated macrophages were found in the injured joint, while cartilage loss and subchondral bone changes were also seen following LPS administration prior to injury (53). These findings have added further support to the concept that overall inflammatory status at the time of injury could also influence eventual disease severity. Viewed from a different perspective, this also suggests that mechanobiological responses may vary depending on overall inflammatory state of a given tissue, at the time of injury. This once again highlights the intersections of immune function and status with mechanobiology in this scenario.

## Mechanobiological Responses Are Linked to Inflammation and Mitochondrial Dynamics

Independent of the inflammatory responses to injury, although possibly involved in their initiation, the mechanobiological properties of joint tissues are likely to be involved in linking injury with subsequent disease. Furthermore, as with inflammatory stimulus, mechanical forces have been shown to be capable of modulating mitochondrial function (54, 55). It is clear that mechanical forces are inherent in joint function, and that joint (and general) health can benefit greatly from moderate mechanical loading (i.e. exercise, which is viewed, in many quarters, as being a highly effective tool/treatment to combat OA) (56). Mechanical stresses that exercise generates are manifested at the cellular level in a variety of ways. For example shear stress (which can be defined as a force that causes deformation in the plane of the surface) is generated within the joint by the flow of synovial/interstitial fluid across, and through, cartilage tissue (57). Also hydrostatic pressures can be generated in the joint from the compression and expansion of the ECM (58). However, when mechanical forces exceed a certain threshold (i.e. injury) joint damage, can occur which is in turn linked to consequent chronic diseases like PTOA (56). Surprisingly, precisely which cell type in the joint predominates in the mechanobiological response is not currently clear. Cells in synovial, bone and cartilage tissue itself have all been found to be highly mechanosensitive (i.e. show altered biochemical activities solely due to changes in their physical/mechanical environment). Mechanotransduction in human primary synovial fibroblasts was demonstrated experimentally by application of uniaxial cyclical stretch tests (using a membrane deformation assay), resulting in cell orientation, and cytoskeletal alignment, changes perpendicular to the applied stress. Those studies used the same system to show that stretch testing also resulted in significant increases in intracellular calcium [Ca^2+^]. This response was then blocked using nonspecific calcium channel blockers [Ruthenium Red (RR)] (59). While this method did not directly assess the level of mitochondrial involvement, it is interesting to note that RR has also been shown to block mitochondrial Ca^2+^ uptake in other scenarios (60), which points to a potential link between these processes. In a similar study, synovial fibroblasts were exposed to shear stresses by application controlled fluid flow in a specialized bioreactor culture system, and again were found to be highly sensitive to changes in shear stress. This study also demonstrated a robust calcium signalling response involving both external and internal calcium sources (61), again highlighting the importance of mechanobiology and mitochondrial dynamics in joint tissue responses to injury.

As described above, in addition to synovial fibroblasts the intimal synovial membrane also contains resident macrophage like synoviocytes (MLS), under normal healthy conditions. The total number of these cells present in the synovium increases dramatically after injury, when circulating monocytes are recruited from the vasculature to the subintimal layer (62, 63). As with synovial fibroblasts, macrophages have also been found to be responsive to mechanical stimulus. A recent study reported that macrophages express high levels of Piezo1, a mechanically activated calcium channel (64). This study was focused on lung inflammation, where hydrostatic pressures predominate in the cellular microenvironment. Thus, by applying hydrostatic pressure to macrophages, Piezo1 was shown to facilitate calcium influx, driving activation of AP-1, which in turn causes release of Endothelin 1 and stabilization of HIF-1α. This upregulates a spectrum of pro-inflammatory genes, including IL-1β. While this paper focused on hydrostatic pressure within the respiratory system, it nonetheless suggests that this mechanistic response may be preserved in macrophages at other sites, and in other tissues. Also, while the role of mitochondria in the response was not addressed these studies, it is well documented that mitochondria can stabilise HIF-1α *via* the production of mitochondrial ROS pointing to a potential mitochondrial connection (65). TRPV4 is another calcium influx channel which has been shown to be involved in mechanotransduction and oxidative stresses responses in macrophages. This channel becomes activated in macrophages following a range of stimuli including mechanical stretch (66). Stimulating this channel results in an increase in mitochondrial membrane potential, *via* as yet unknown mechanisms, as well as greater ROS and nitric oxide production. While this was shown to be relevant to the pro-inflammatory response induced by hydrostatic pressure, it is likely that a similar mechanism may occur in the mechanical environment of the joint.

Cartilage cells themselves have also been shown to be highly sensitive to mechanical stimulus and damage. Intriguingly, Piezo channels 1 and 2 (and TRPV4) were again found to be central and were identified in human chondrocytes where they were shown to be intimately involved in mechanotransduction and injury responses (67, 68). Their expression was also significantly increased in human osteoarthritic cartilage. Increased Piezo1 expression in chondrocytes resulted in a feed-forward mechanism whereby it induced excess intracellular Ca^2+^, at baseline and in response to mechanical deformation (69) Using a bioreactor system with human chondrocytes isolated from end stage OA cartilage Delco et al. (51) also demonstrated acute cartilage responses to mechanical loading. Within 2 hours of stimulus/injury the endogenous mitochondrial respiratory function was impaired and membrane depolarisation had occurred. Targeting of mitochondrial potential, capacity, and membrane polarisation early in the post-injury period may lead to discovery of factors that drive cartilage degradation after injury. It may become possible to intervene, early after injury, using targeted mitochondrial therapeutics to rescue the joint from significant long-term damage. The extent/severity of mechanical force/impact which the cartilage undergoes is also important in determining the eventual outcome of disease - adding yet further complexity to understanding this injury/disease system. Bonnevie et al. (70) reported that mechanically impacting cartilage tissue, in the stress range of 15-20 MPa, results in significant chondrocyte death. It has also been shown that impact forces below this range can induce matrix breaches (6), depolarisation of mitochondrial membranes (71), and catabolic cellular responses (72) and upregulation of matrix degradation enzymes including MMP and ADAMTS.

Mechanical loading of cartilage, above a certain threshold level has also been shown to create an imbalance in mitochondrial superoxide levels (9). For example, delivery of a permeable antioxidant ascorbyl 6-palmitate 2-phosphate (APPS), a derivative of vitamin C, to the site of injury was shown to effectively suppress the response and reduce cartilage degeneration in mice (9). Elsewhere, repeated mechanical overloading of cartilage was shown to produce an oxidant-dependant state of mitochondrial dysfunction in chondrocytes (73). Furthermore it was shown that this damaging outcome could be rescued *via* introduction of free radical scavengers or disruptors of the electron transport chain (ETC), such as rotenone (inhibitor of complex 1 of the ETC) (74). In a related, and very relevant, study using a porcine model of PTOA, targeting mitochondrial responses following mechanical injury had favourable outcomes in terms of reducing disease severity at six months post-injury (75). Injury-induced changes to the ETC in chondrocytes has been linked with greater oxidative damage and ultimately cell death (12). This study used amobarbital to inhibit chondrocyte electron transport or N-acetylcysteine (NAC) to inhibit oxidative stress further downstream (75). Both treatments resulted in maintenance of proteoglycan content, decreased histological severity, and more normalised chondrocyte metabolic function 6 months post injury. These studies once again show that mitochondrial function is critical for maintenance of cellular energy production *via* the gradient created in the ETC in joint tissues. Pathogenic unfolding of membrane cristae and loss of membrane polarisation are characteristic of diseases in many tissues, but it is interesting to note that the same has recently been shown to be true in OA (76). These studies also support the potential application of antioxidants and targeting chondrocyte mitochondrial metabolism after injury to mediate PTOA and promote healthy cartilage (75). Ultimately, while this work is still at a relatively early stage, and biological means of repairing damage to cartilage after injury remains elusive; determining the role of mechanotransduction in damaged joint tissues, and the intersection this has with mitochondrial function, inflammation and PTOA, may reveal exciting possibilities for new therapies and targets in the joint.

## Therapeutic Potential

Taking these findings together, the emerging theme is that mitochondria, through a number of mechanisms, are extremely important for joint injury and disease. Therefore the next question is whether we can target this organelle and its function for therapeutic gain in the treatment of joint disease. It has been demonstrated that therapies aimed at mitochondrial repair, for example Szeto-Schiller (SS) peptides developed by Szeto et al. (77–79) – in particular SS-31, are protective to mitochondria after impact and subsequent degeneration. This effect is achieved *via* targeting the permeability of the mitochondrial membrane and production of ROS (77). Specifically, SS peptides work by interacting with cardiolipin and cytochrome c (78) thus producing an antioxidant effect on the inner mitochondrial membrane (80). These peptides have also been shown to protect chondrocyte viability by prevention of cytochrome c release and induction of apoptotic cascade (80). Moreover, they have the ability to preserve cartilage integrity and chondrocyte cell viability after impact in an *ex vivo* model (81). Investigation of mitochondrial therapy at this level suggests that compounds which target these pathways may have great utility in prevention of the onset of PTOA, even in cases where administration occurs up to 12 hours post-injury (81).

Another potential strategy involves targeting mitochondrial ROS production directly. A recent study used the antioxidant, Licochalcone A (Lico A), to limit NLRP3 inflammasome induced damage to chondrocytes *in vitro* and in a surgical model of OA (40). Those studies showed that Lico A can ameliorate chondrocyte damage and death by promoting the NRF2/HO-1 axis to limit NF-kB activation during injury. Further studies have identified the therapeutic potential of nanoparticles to successfully deliver and retain anti-oxidant agents to chondrocytes, and cartilage protection. While promoting the use of antioxidants, these studies also highlighted the viability of NLRP3 inhibitors in OA. A potent inhibitor of NLRP3, MCC950, has also come to prominence in the wider field of immunology, and has been shown to be both safe and effective in limiting NLRP3 activity in human models of disease (82). While joint diseases such as gout, which involves direct activation of NLRP3 within the joint may be the first targets of such drugs, there is also great potential for them to provide benefits as a first line, early intervention, strategy in PTOA prevention.

## Conclusion

In conclusion strong links have recently emerged between mechanobiology, mitochondrial function, inflammation/tissue-damage in skeletal joint pathologies. As or understanding of these links are further developed, they combine to form new paradigms for therapeutic intervention, particularly at early stages post-injury, to prevent the subsequent development of chronic PTOA.

## Author Contributions

LF and JE contributed equally to writing and reviewing the manuscript. OK and AC developed the initial idea for the topic and critically evaluated the manuscript through each of the writing stages. All authors contributed to the article and approved the submitted version.

## Funding

This work was funded by the following awards: a Science Foundation Ireland [Career Development Award (17/CDA/4699)] to OK, a Science Foundation Ireland [Career Development Award (17/CDA/4688)] and an Irish Research Council Laureate Award (IRCLA/2017/110) to AC, and LF was funded by an Irish Research Council Government of Ireland Postgraduate Scholarship GOIPG/2018/2752.

## Conflict of Interest

The authors declare that the research was conducted in the absence of any commercial or financial relationships that could be construed as a potential conflict of interest.

## Publisher’s Note

All claims expressed in this article are solely those of the authors and do not necessarily represent those of their affiliated organizations, or those of the publisher, the editors and the reviewers. Any product that may be evaluated in this article, or claim that may be made by its manufacturer, is not guaranteed or endorsed by the publisher.
